# The Identification and Validation of Hub Genes Associated with Acute Myocardial Infarction Using Weighted Gene Co-Expression Network Analysis

**DOI:** 10.3390/jcdd9010030

**Published:** 2022-01-17

**Authors:** Junqiang Xue, Lu Chen, Hao Cheng, Xiaoyue Song, Yuekai Shi, Linnan Li, Rende Xu, Qing Qin, Jianying Ma, Junbo Ge

**Affiliations:** Shanghai Institute of Cardiovascular Diseases, Department of Cardiology, Zhongshan Hospital, Fudan University, Shanghai 200031, China; 19211210020@fudan.edu.cn (J.X.); 20111210132@fudan.edu.cn (L.C.); 19111210041@fudan.edu.cn (H.C.); 18211210013@fudan.edu.cn (X.S.); 20211210004@fudan.edu.cn (Y.S.); 21211210007@fudan.edu.cn (L.L.); xu.rende@zs-hospital.sh.cn (R.X.); qin.qing@zs-hospital.sh.cn (Q.Q.); jbge@zs-hospital.sh.cn (J.G.)

**Keywords:** hub genes, acute myocardial infarction, WGCNA, GSEA, bioinformatics

## Abstract

Acute myocardial infarction (AMI), one of the most severe and fatal cardiovascular diseases, remains the main cause of mortality and morbidity worldwide. The objective of this study is to investigate the potential biomarkers for AMI based on bioinformatics analysis. A total of 2102 differentially expressed genes (DEGs) were screened out from the data obtained from the gene expression omnibus (GEO) database. Weighted gene co-expression network analysis (WGCNA) explored the co-expression network of DEGs and determined the key module. The brown module was selected as the key one correlated with AMI. Gene Ontology and the Kyoto Encyclopedia of Genes and Genomes pathway enrichment analyses demonstrated that genes in the brown module were mainly enriched in ‘ribosomal subunit’ and ‘Ribosome’. Gene Set Enrichment Analysis revealed that ‘TNFA_SIGNALING_VIA_NFKB’ was remarkably enriched in AMI. Based on the protein–protein interaction network, ribosomal protein L9 (RPL9) and ribosomal protein L26 (RPL26) were identified as the hub genes. Additionally, the polymerase chain reaction (PCR) results indicated that the expression levels of RPL9 and RPL26 were both downregulated in AMI patients compared with controls, in accordance with the bioinformatics analysis. In summary, the identified DEGs, modules, pathways, and hub genes provide clues and shed light on the potential molecular mechanisms of AMI.

## 1. Introduction

According to the updated data of Heart Disease and Stroke Statistics (2020 Edition), cardiovascular disease (CVD) remains the leading cause of death globally, leading to 17.8 million (95% CI, 17.5–18.0 million) deaths worldwide in 2017, which remarkably aggravates the global health burden [1]. Acute myocardial infarction (AMI), one of the most severe cardiovascular diseases, is characterized by the sudden depletion of blood flow to the myocardium, with high morbidity and mortality. It affected an estimated 7.29 million people in 2015 [2]. With the increase in the life expectancy of the population, effective strategies to prevent and treat AMI have become more urgent than ever. Studies have shown that the immune system has a pivotal impact on the pathophysiology of coronary artery disease [3]. As the core component of the immune system, peripheral blood consisting of immune cells, such as lymphocytes, monocytes, and neutrophils, likely regulates the process of AMI. Recent evidence that neutrophil amplifies granulopoiesis after myocardial infarction [4], suggests that identifying the change in peripheral blood during AMI may pave novel therapeutic avenues for AMI.

Gene chips, a high-sequence approach to detecting transcriptome changes, have been widely applied in most disease research, including CVD in the post-genome era [5]. Attributed to a mass of expression data from gene chips, the correlation between genes and disease can be readily investigated.

Weighted gene co-expression network analysis (WGCNA) is an effective technique for processing transcriptome data, as it clusters tightly connected genes into different modules and explores the correlation between modules and traits of interest; it does not only focus on the differentially expressed genes [6]. Scale-free co-expression network analysis using WGCNA has been conducted in most disease research [7,8,9].

In this study, we re-analyzed the gene expression profile of GSE with 14 AMI patients and 10 controls. The differentially expressed genes were obtained by the ‘limma’ package in R software. WGCNA was conducted to build a gene co-expression network. Gene ontology and KEGG pathway enrichment analysis were performed in the key module. Hub genes in the key module were determined and validated by quantitative RT-PCR.

## 2. Materials and Methods

### 2.1. Data Sources

**Figure 1:**
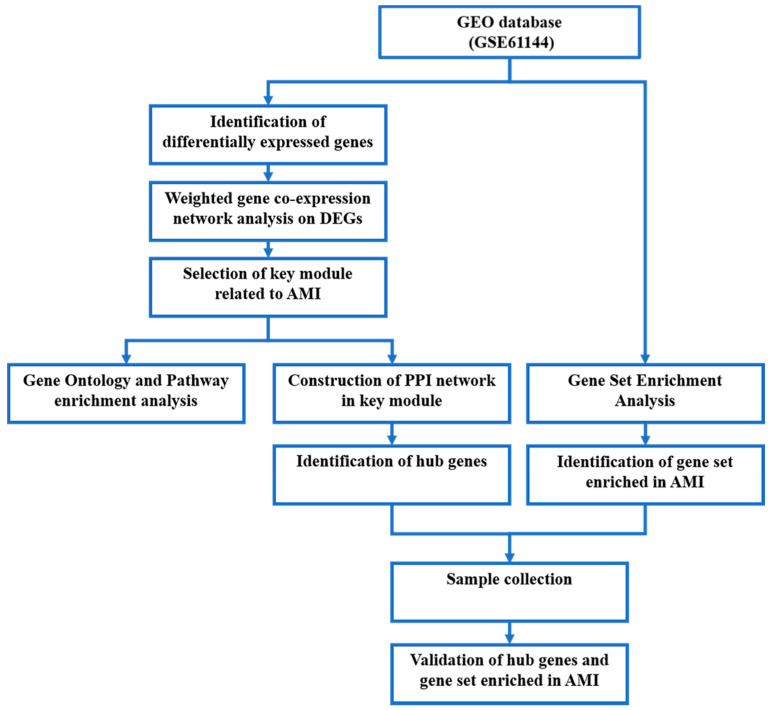
Workflow chart of the present study.

A workflow of the present study is presented in Figure 1. The mRNA expression profile microarray GSE, deposited by Park et al. [10], was downloaded from the free public database Gene Expression Omnibus (GEO). It contains peripheral blood from AMI patients and controls. The data were processed on platform GPL6106 (Sentrix Human-6 v2 Expression BeadChip, Bethesda, MD, USA), including 24 samples, 14 from AMI patients and 10 from controls.

### 2.2. Identification of Differentially Expressed Genes

Data quality checking and normalization with log transformation were first performed to eliminate any batches. The ‘limma’ package [11] in R software (v4.1.1, Vienna, VIE, Austria) was employed to screen DEGs between the AMI and control group. An adjusted *p*-value < 0.05 was set as the threshold criterion for statistical significance. The volcano map of DEGs was plotted in R software. The Heatmap package in R software was utilized to visualize the top 50 DEGs.

### 2.3. Weighted Gene Co-Expression Network Analysis

WGCNA [6] was conducted to build the co-expression network in DEGs based on the scale-free topology criteria. First, all DEGs were analyzed using the WGCNA package in R software, and the soft thresholding power was determined. Next, the weighted co-expression network was constructed, and DEGs were clustered into several modules with different color labels. The correlation between each module and AMI or controls was then explored. The module most correlated with AMI was regarded as a key module for further enrichment analysis.

### 2.4. Gene Ontology and Pathway Enrichment Analysis

Metascape [12] (v3.5, San Diego, CA, USA)is an efficient approach to investigating the potential biological process of transcriptome and genome data and the associated pathways. Gene ontology (GO) [13] analysis (containing the biological process, cellular component, and molecular function) and KEGG [14,15,16] pathway analysis were both conducted in the key module using Metascape. The function and pathway terms were retrieved for further visualization in R software.

### 2.5. Gene Set Enrichment Analysis

Gene Set Enrichment Analysis (GSEA) [17] was applied to detect whether the enrichment of the KEGG pathway is statistically significant in AMI patients and controls. Transcriptome data were imported into the GSEA desk application strictly according to the website instruction. Both *p* < 0.05 and FDR < 0.25 were considered as the criteria for the significant gene sets.

### 2.6. Protein–Protein Interaction Network and Hub Gene Identification

The Search Tool for Retrieval interacting Genes (STRING) v11.0 [18] online database was employed to build the protein–protein interaction (PPI) network in the key module. The PPI network was then visualized by Cytoscape software [19]. Furthermore, significant modules in the PPI network were obtained by the molecular complex detection (MCODE) [20] plug-in, with the degree cutoff = 2, max depth = 100, and k-score = 2. Cytohubba [21], a plug-in in Cytoscape software, was used to determine the hub genes.

### 2.7. Putative Signaling Pathways Involving Hub Genes and GO Analysis

‘GeneMania’ [22] is a comprehensive web-based tool that indexes 2830 interaction networks mapped to 166,691 genes from 9 organisms, which helps predicate the function of preferred genes. Identified hub genes were imported into the database ‘Genemania’ to establish a putative protein–protein interaction work. The total genes derived from the network then conducted GO analysis using the Metascape tool (v3.5, San Diego, CA, USA). The results were processed and visualized in R software.

### 2.8. Sample Collection

This study was approved by the Medical Ethics Committee of Zhongshan Hospital, Fudan University (approval number: B2021-073), which complied with the Declaration of Helsinki. All subjects signed written informed consent. A total of fourteen AMI patients and eight controls were enrolled in the study. The diagnosis of AMI was in line with the Fourth Universal Definition of Myocardial Infarction (2018) [23]. AMI is diagnosed when there is clinical evidence of acute myocardial ischemic and the rise or fall of cTnT values with at least one value exceeding the 99th percentile upper reference limit, followed by at least one of the followings: (1) symptoms of myocardial ischemia, (2) changes on ECG indicating new ischemia, (3) development of pathological Q waves on ECG, (4) evidence of new loss of viable myocardium or new regional wall motion abnormality by imaging, and (5) coronary thrombus by angiography or autopsy. Subjects with no symptoms of myocardial ischemic, no ischemic changes on ECG, or no stenosis in the coronary angiography were regarded as the controls. Peripheral blood was collected as coronary angiography was performed.

### 2.9. RNA Extraction and Quantitative RT-PCR

Total RNA from peripheral blood was extracted using the UNlQ-10 Column Trizol Total RNA Isolation Kit (Sangon Biotech, Shanghai, China) according to the manufacturer’s instructions. The NanoDrop 2000 Spectrophotometer (Thermo Fisher Scientific, Waltham, MA, USA) was utilized to check the concentration and purity of the extracted RNA, with the A260/A280 between 1.8 and 2.0. The cDNA synthesis was conducted using Hifair Ⅲ 1st Strand cDNA Synthesis SuperMix (Yeasen Biotech, Shanghai, China). Using β-actin as a reference, we performed quantitative RT-PCR with Hieff qPCR SYBR Green Master Mix (Yeasen Biotech, Shanghai, China) in the QuantStudio 6 Flex system (Thermo Fisher Scientific, Waltham, MA, USA). Primer sequences (Sangon Biotech, Shanghai, China) for reference and candidate genes are shown in Table 1. The 2^−ΔΔCt^ method was applied to calculate the relative expression level of mRNA.

### 2.10. Statistical Analysis

The SPSS (v23.0, Armonk, NY, USA) and GraphPad Prism 9 software (San Diego, CA, USA) were employed to analyze the data. Normal distribution measurement data were displayed as mean ± SD, while abnormal measurement data as median (25th–75th percentile) by SPSS 23.0. The statistically significant differences between the AMI group and controls were examined by Student’s *t*-test or Mann–Whitney U test in GraphPad Prism 9 (San Diego, CA, USA). The construction of receiver operator characteristic (ROC) curve and the calculation of the area under the ROC curve (AUC) were finished in GraphPad Prism 9. The statistical significance was set as *p* < 0.05.

## 3. Results

### 3.1. Identifications of DEGs

**Figure 2:**
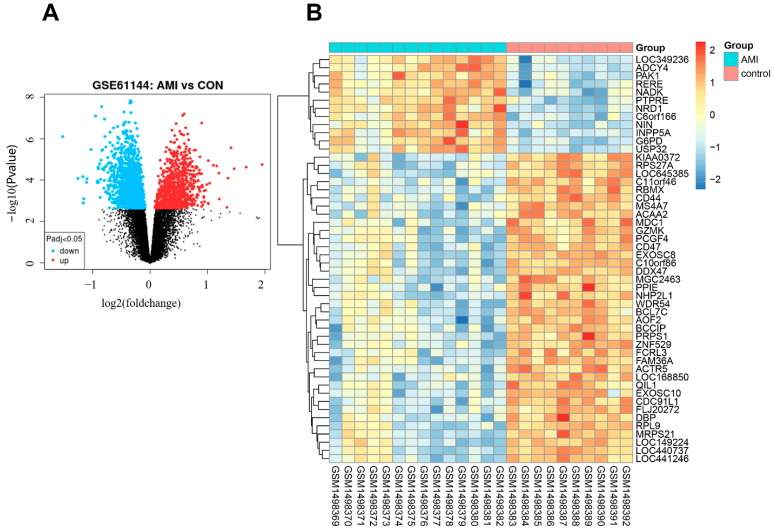
Expression profile of DEGs. (**A**) Volcano map of DEGs expression levels. (**B**) Heatmap of top 50 DEGs.

PCA plots before and after batch correction are shown in Figure S1. A total of 2102 differentially expressed genes were identified, with an adjusted *p*-value < 0.05 between AMI patients and controls. Of these DEGs, 781 genes were upregulated and 1321 genes were downregulated in AMI. The volcano map of DEGs is shown in Figure 2A. The heatmap for the top 50 DEGs is displayed in Figure 2B and Table S1.

### 3.2. GWCNA Analysis

**Figure 3:**
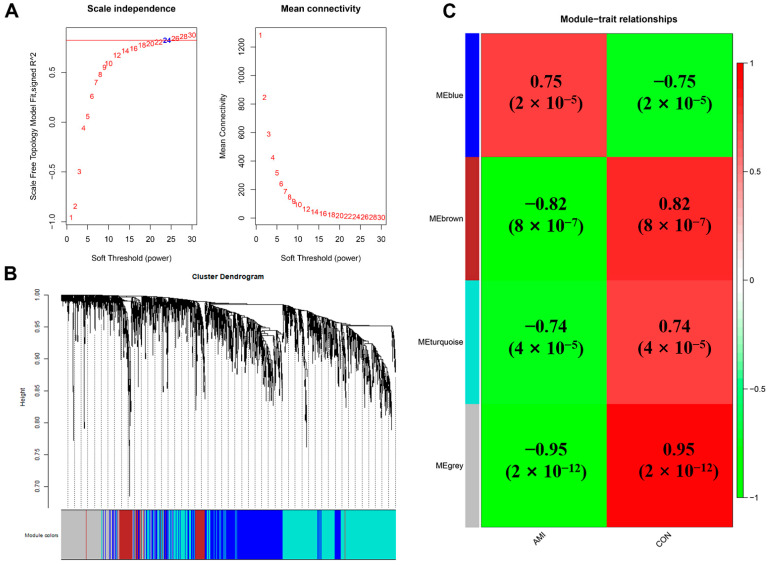
WGCNA of DEGs. (**A**) Estimation of the soft thresholding value for a scale-free co-expression network. (**B**) Cluster dendrogram of all DEGs. (**C**) Correlation between each module and AMI patients or controls.

The 2102 identified DEGs were further processed with the GWCNA package in R software, and a scale-free co-expression network (scale-free R^2^ > 0.8) was established using a soft thresholding power of 24. The soft thresholding power β was set at 24 in the subsequent analysis, because the scale independence reached 0.823 (Figure 3A) and had a relatively good average connectivity. The DEGs were clustered into four modules, blue, brown, turquoise, and grey, with a minimal module size ≥30. The cluster dendrogram of the DEGs is shown in Figure 3B. The correlation between each module and AMI was calculated and plotted (Figure 3C). The results indicated that brown (−0.82, *p* < 0.0001) and blue (0.75, *p* < 0.0001) were the most negative and positive modules related to AMI, respectively. Herein, the brown module, including 196 DEGs, was considered as a key module correlated to AMI. The top 50 DEGs in the brown module are shown in Table S2.

### 3.3. Functional Enrichment Analysis

**Figure 4:**
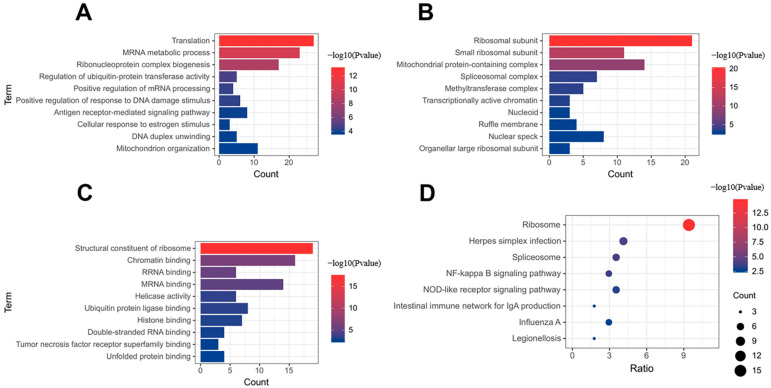
Gene ontology and KEGG enrichment analysis. (**A**) Biological process. (**B**) Cellular component. (**C**) Molecular function. (**D**) KEGG enrichment analysis.

The 196 DEGs in the brown module were used for gene ontology and pathway enrichment analysis with the Metascape tool. GO analysis revealed that DEGs in the brown module were enriched in 154 biological processes (BP), 40 cellular components (CC), and 26 molecular functions (MF). The top 10 BP, CC, and MF are shown in Figure 4A–C. The GO category showed that ‘translation,’ ‘ribosomal subunit,’ and ‘structural constituent of ribosome’ were markedly enriched in the brown module. KEGG analysis demonstrated that the brown module was involved in 22 pathways, including ‘Ribosome,’ ‘Herpes simplex infection,’ and ‘Spliceosome’ (Figure 4D).

### 3.4. GSEA Analysis

**Figure 5:**
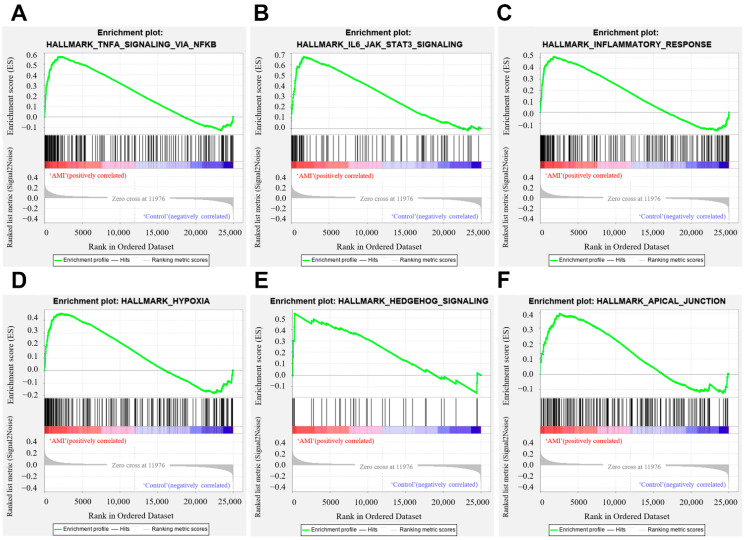
Gene set enrichment analysis. (**A**) Enrichment plot of ‘TNFA_SIGNALING_VIA_NFKB’ with enrichment score 0.57, FDR *q*-value 0.0. (**B**) Enrichment plot of ‘IL6_JAK_STAT3_SIGNALING’ with enrichment score 0.67, FDR *q*-value 0.0. (**C**) Enrichment plot of ‘INFLAMMATORY_RESPONSE’ with enrichment score 0.51, FDR *q*-value 0.004. (**D**) Enrichment plot of ‘HYPOXIA’ with enrichment score 0.46, FDR *q*-value 0.0025. (**E**) Enrichment plot of ‘HEDGEHOG_SIGNALING’ with enrichment score 0.54, FDR *q*-value 0.05. (**F**) Enrichment plot of ‘APICAL_JUNCTION’ with enrichment score 0.40, FDR *q*-value 0.047.

The distribution of the pathway gene sets on all gene expression data from the AMI patients and controls was explored using the GSEA software. The results showed that 30/50 gene sets were upregulated in the AMI patients, while 24 gene sets were significantly enriched with FDR < 25%. In the controls, 20/50 gene sets were upregulated, and 11 gene sets were highly enriched with FDR < 25%. ‘TNFA_SIGNALING_VIA_NFKB’ was remarkably enriched in the AMI group, with an enrichment score of 0.57 (Figure 5A), suggesting that ‘TNFA_SIGNALING_VIA_NFKB’ may play a pivotal role in the pathophysiology of AMI. The top six gene sets were shown in Figure 5A–F.

### 3.5. PPI Network Construction, Modular Analysis, and Hub Gene Analysis

**Figure 6:**
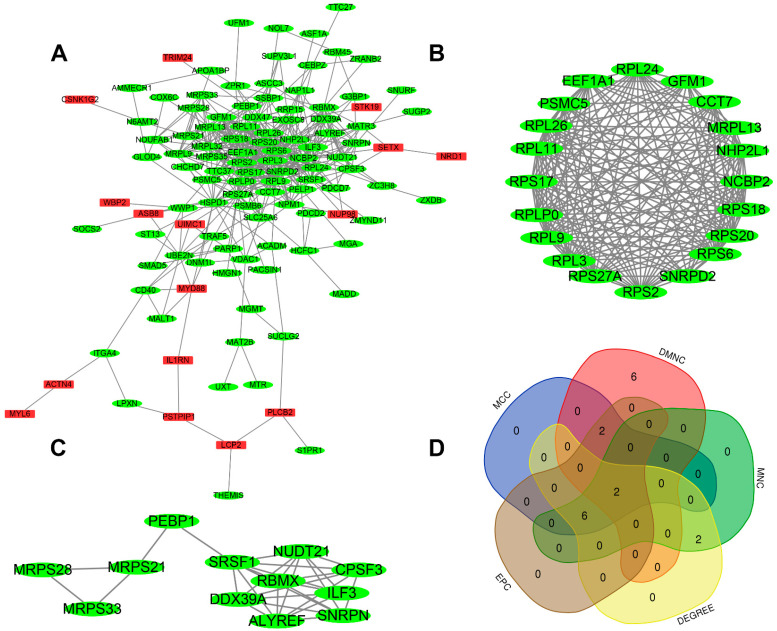
PPI network and hub gene. (**A**) PPI network. Genes in red represent upregulation; genes in green represent downregulation. (**B**,**C**) The two most significant modules. (**D**) The overlapped hub genes from different algorithms.

To explore the interaction of genes in the brown module, a protein–protein interaction network was constructed using the STRING database. Then, 0.4 was set as the threshold as the minimum required interaction score for constructing the STRING PPI network. As is shown in Figure 6A, the PPI network comprised 114 nodes and 526 edges. There were 16 upregulated genes and 98 downregulated genes in the PPI network. Using the MCODE plug-in in Cytoscape, two modules were determined. Module 1 (score = 17.789) included 20 nodes and 169 edges (Figure 6B), and module 2 (score = 5.454) consisted of 12 nodes and 30 edges (Figure 6C). The top ten hub genes obtained by five algorithms, MCC, DMNC, MNC, Degree, and EPC, in the cytohubba plug-in, are shown in Table 2. The overlapped hub genes among the five algorithms were verified by a Venn diagram (Figure 6D), including RPL9 and RPL26.

### 3.6. Construction of Putative RPL9 and RPL26 Protein–Protein Interaction Network and GO Analysis

**Figure 7:**
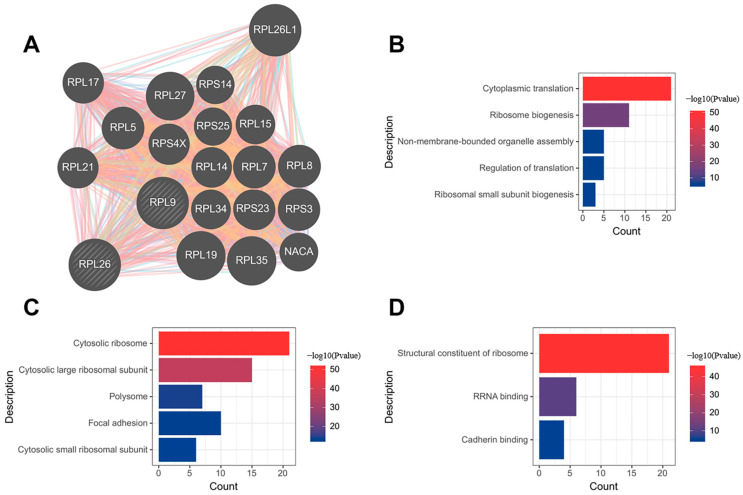
Putative RPL9 and RPL26 protein–protein interaction network and GO analysis. (**A**) PPI network. (**B**) Biological process. (**C**) Cellular component. (**D**) Molecular function.

By employing the tool ‘GeneMania’, we constructed a putative protein–protein interaction network of 22 genes involving hub genes RPL9 and RPL26. The PPI network contained 2823 total links as is shown in Figure 7A. Gene ontology analysis on the network showed that 22 genes were mostly enriched in 5 biological processes, 5 cellular components, and 3 molecular functions, which are visualized in Figure 7B–D. RPL9 and RPL26, as the hub genes of AMI, putatively participated in the ‘cytoplasmic translation’ and ‘ribosome biogenesis’, indicating that intense ribosomal changes can occur in the pathogenesis of AMI.

### 3.7. Baseline Characteristics of Study Subjects

Twenty-two participants were recruited in the present study and were separated into two groups (AMI (*n* = 14) and controls (*n* = 8)). The average age of the AMI group was 60.714, while that of controls was 60.571. The two groups were well matched in terms of age and gender. The demographic, clinical features, medications, and laboratory data of all participants are shown in Table 3.

### 3.8. Validation of the Hub Genes

**Figure 8:**
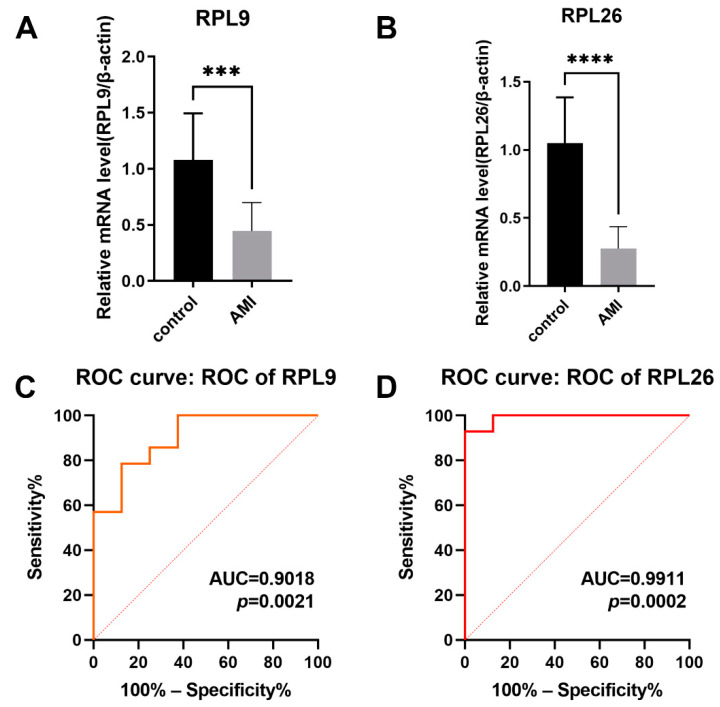
Validation of hub genes. (**A**) Relative mRNA level of RPL9 in controls vs. AMI patients. (**B**) Relative mRNA level of RPL 26 in controls vs. AMI patients. (*** *p* < 0.001, **** *p* < 0.0001). (**C**) ROC curve for RPL9. (**D**) ROC curve for RPL26.

The transcriptional changes of overlapped hub genes RPL9 and RPL26, were detected in the peripheral blood from the AMI patients and controls by quantitative RT-PCR. The results indicated that the expression levels of RPL9 and RPL26 were both decreased in the AMI group in comparison with those in controls (Figure 8A,B), which was in line with the bioinformatics analysis. To evaluate the capability of RPL9 and RPL26 to distinguish the AMI group from controls, ROC curves were performed. According to the results, the AUCs of RPL9 and RPL26 were 0.9018 (95% CI 0.7712–1.000; *p* = 0.0021) and 0.9911 (95% CI 0.9628–1.000; *p* = 0.0002), respectively, showing that the identified hub genes RPL9 and RPL 26 demonstrated a powerful discrimination capability as potential biomarkers for AMI.

### 3.9. Validation of the Gene Set ‘TNFA_SIGNALING_VIA_NFKB’

**Figure 9:**
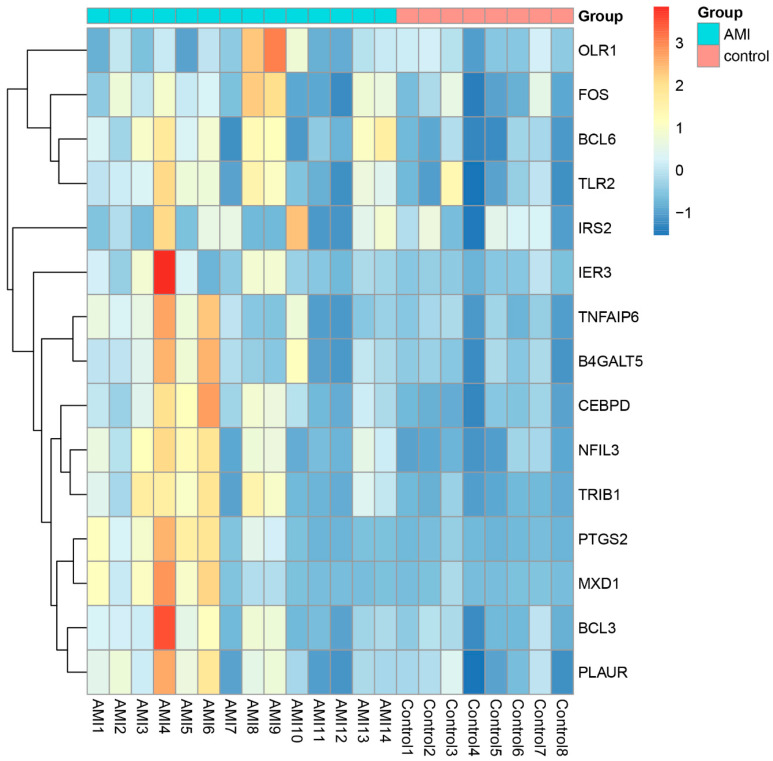
Validation of the gene set ‘TNFA_SIGNALING_VIA_NFKB’. Heatmap of the key genes of gene set ‘TNFA_SIGNALING_VIA_NFKB’ in the peripheral blood of AMI and controls.

The enrichment of gene set ‘TNFA_SIGNALING_VIA_NFKB’ was experimentally validated by detecting the expression level of key genes of ‘TNFA_SIGNALING_VIA_NFKB’ signaling in the peripheral blood from the recruited patients. The results in Figure 9 indicate that the expression level of most key genes are elevated in the AMI group. The findings provide experimental evidence to demonstrate the molecular determinants of ‘TNFA_SIGNALING_VIA_NFKB’ signaling involved in AMI.

## 4. Discussion

Acute myocardial infarction remains one of the leading causes of death worldwide [24]. Owing to the sudden shortage of blood supply to the coronary artery and following the depletion of energy and nutrients, the myocardium dies quickly. AMI patients might experience severe chest pain, weakness or lightheadedness, arm or shoulder discomfort, shortness of breath, and jaw, neck, or back discomfort [1]. Without timely intervention, most people may die or survive with a variety of disabilities. Although reperfusion strategies and pharmacological treatments save many lives, AMI is still a global burden [24]. The etiology, effective diagnostic markers, and therapeutic strategies remain to be fully elucidated.

In this study, we performed a WGCNA on mRNA expression profile GSE6114 downloaded from the GEO database. With this novel approach, all 2102 differentially expressed genes obtained by the ‘limma’ algorithm were clustered into four modules. Then, 196 genes in the brown module were found to be the most closely related to the AMI (correlation score = −0.82, *p* < 0.0001). These genes were mainly enriched in ‘translation,’ ‘ribosomal subunit,’ ‘structural constituent of ribosome,’ and ‘ribosome.’ GSEA showed that the gene set ‘TNFA_SIGNALING_VIA_NFKB’ was markedly enriched in the AMI group. Two hub genes, RPL9 and RPL26, were determined based on the PPI network. The expression levels of RPL9 and RPL26 and the gene set ‘TNFA_SIGNALING_VIA_NFKB’ were validated by quantitative RT-PCR.

According to the GO analysis, the BP term ‘translation’ mainly affected protein synthesis, which otherwise resulted in the production of dysfunctional proteins. ‘Translation’ has been previously reported correlated to AMI [25]. Donko et al. also stated that the unique genes in degenerative heart disease encoded proteins in the 80 S ribosome complex (term ‘translation’) [26]. The CC term ‘ribosomal subunit’ and the MF term ‘structural constituent of ribosome’ were both notably associated with the ribosome formation, which is an essential part of protein translation [27]. KEGG analysis revealed that the ‘ribosome’ pathway can play a marked role in peripheral blood following AMI. Interestingly, Bittman et al. discovered that altered genes in patients with coronary heart disease were mainly related to ‘ribosome’ while making recreation music [28]. Li et al. showed that ‘ribosome’ was correlated with hypertrophic cardiomyopathy [29]. Although the investigation of ‘ribosome’ on AMI is limited, the previous studies of other cardiovascular diseases provide clues that ‘ribosome’ might be a good index for further research in AMI. By performing the GSEA analysis on the gene profile of GSE, we obtained lots of gene sets highly enriched in the AMI group. Among them, ‘TNFA_SIGNALING_VIA_NFKB’ with an enrichment score of 0.57, was experimentally validated. Briefly, ‘TNFA_SIGNALING_VIA_NFKB’ refers to the genes regulated by NF-kB in response to TNF. This gene set comprises 182 genes, including the well-known tumor necrosis factor alpha (TNF-α), nuclear factor kappa B (NFKB), and other inflammatory cytokines [17]. TNF-α has been proved to be enhanced in MI [30]. Multiple studies showed that TNF-α inhibitor reduced the infarct area [31]. However, the administration of low-dose TNF-α prior to ischemic reperfusion in animal models led to a reduction of infarct area, suggestive of a precondition effect [32]. NFKB is a homo- or hetero-dimeric complex formed by the Rel-like domain-containing proteins RELA/p65, RELB, NFKB1/p105, NFKB1/p50, REL, and NFKB2/p52, with the p65–p50 complex as the most abundant. NFKB participates in many biological processes, such as inflammation, immunity, differentiation, cell growth, tumorigenesis, and apoptosis. The crosslink between NFKB and cardiovascular diseases has also been built [33]. Emerging evidence shows that intense inflammatory responses occur during AMI. We are delighted to find that some important inflammatory cytokines are enriched in AMI, in accordance with the previous findings.

Compared with previous studies, our work provides a unique insight into the underlying pathogenesis of AMI. Liu et al. obtained three microarray datasets (GES, GSE, and GSE) and screened out three genes, FGFBP2, GFOD1, and MLC1, as potential markers for the diagnosis of AMI [34]. Liu et al. performed a WGCNA on GSE4648 to determine the genes correlated with AMI, ferroptosis, and hypoxia. They clarified the possibility of 10 hub genes (Atf3, Ptgs2, Cxcl1, Socs3, Hspa1 b, Selp, Cxcl2, Il1 b, Myd88, and S100 a8) as diagnostic markers for AMI utilizing bioinformatic and experimental methods [35]. By contrast, Zhang et al. separately analyzed the hub genes associated with STEMI and NSTEMI [36]. They found that Aqp1, Armcx1, Gsta4, Hist3 h2 a, and Il17 as hub genes of STEMI were mainly enriched in cell membrane signal transduction, while Olr1, Nap1 l3, Gfer, Dohh, Crispld1, and Ccdc8 b as hub genes of NSTEMI were markedly related to energy metabolism [36]. Intriguingly, Xie et al. inferred that four genes (FN1, CD34, LPL, and WWTR1) were capable of distinguishing STEMI patients from healthy controls and SCAD [37]. Wang et al. identified 4 hub genes (LILRB2, TLR2, NCF2, and S100 A9) related to AMI based on three databases (GSE, GSE, and GSE) [38]. However, the identification of RPL9 and RPL26 in our study has not been previously investigated to be related to AMI.

Ribosomal proteins (RPs) are structural constituents of the ribosome complex and assemble orderly in concert with cell growth and proliferation [39]. In addition to optimizing the synthesis of proteins, RPs also act as sentinels for the self-evaluation of cellular health [40]. The perturbation of the expression of RPs can directly dysregulate the normal ribosomal function and lead to a range of pathologies. Several RPs have proved to be associated with the development of cardiovascular disease [41,42,43,44]. Cardiomyopathy with the Minute syndrome in *Drosophila melanogaster* is attributed to the haploinsufficiency of the ribosomal protein gene [41]. Ribosome protein L17 suppresses vascular smooth muscle growth and carotid intima formation [43]. Increased plasma protein PCSK9, which has been confirmed to be related to cardiovascular disease, may be mitigated by a small molecule by targeting the 80 s ribosome [44].

Ribosomal protein L9 (RPL9), which has not been widely connected with human disease, can cause deleterious changes in ribosome function and cell metabolism with different variants [45]. Ribosomal protein L26 (RPL26), comprising of 145 amino acids and having a molecular mass of 17,266 Da [46], has previously been reported to regulate the p53 translation with nucleolin [47] or Mdm2 [48]. The knockdown of RPL26 and RPL29 expression ablates the proliferation of human pancreatic cancer PANC-1 cells [49]. According to our results, the expression levels of RPL9 and RPL26 were downregulated in the AMI group, indicating that RPL9 and RPL26 may be cardioprotective during AMI. In combination with the previous study, we suppose that RPL9 and RPL26 can regulate the vitality of the myocardium in the pathological process of AMI. Nevertheless, further research on the role of RPL9 and RPL26 is required to validate the relationship.

According to the ROC curve, the values of the AUCs of RPL9 and RPL26 were 0.9018 and 0.9911, respectively. In general, an AUC of 0.5 indicates no discrimination, 0.7–0.8 is acceptable, 0.8–0.9 is excellent, and over 0.9 is outstanding [50]. Both the AUCs of RPL9 and RPL26 can be regarded as outstanding, indicating the powerful ability of RPL9 and RPL26 to discriminate AMI from the controls. However, the expansion of the sample size is required to validate the efficacy of RPL9 and RPL26 as biomarkers for AMI in the future.

## 5. Conclusions

Conclusively, we re-analyzed the expression profile GSE6114 with WGCNA. Two hub genes in AMI, RPL9, and RPL26 were determined and confirmed by quantitative RT-PCR. To the best of our knowledge, research on the role of RPL9 and RPL26 in AMI remains limited. Our results indicate that RPL9 and RPL26 can participate in the pathophysiology of AMI and serve as potential targets for diagnosis or therapy for AMI.

## Figures and Tables

**Table 1:** Primer sequences for quantitative real-time PCR.

Gene	Primer Sequence (5′→3′)	
RPL26	Forward	ACAACTGTCCACGTAGGCATTCAC
	Reverse	TACTTGGCGAGATTTGGCTTTCCG
RPL9	Forward	TTACACTGGGCTTCCGTTACAAGATG
	Reverse	GCAACACCTGGTCTCATCCGAAC
TNFAIP6	Forward	TTTCTCTTGCTATGGGAAGACAC
	Reverse	GAGCTTGTATTTGCCAGACCG
IRS2	Forward	CGGTGAGTTCTACGGGTACAT
	Reverse	TCAGGGTGTATTCATCCAGCG
B4 GALT5	Forward	TCCTCGCTGCTGTACTTCG
	Reverse	AATGCCTTGGGCTTGCATCA
OLR1	Forward	TTGCCTGGGATTAGTAGTGACC
	Reverse	GCTTGCTCTTGTGTTAGGAGGT
FOS	Forward	CCGGGGATAGCCTCTCTTACT
	Reverse	CCAGGTCCGTGCAGAAGTC
NFIL3	Forward	AAAATGCAGACCGTCAAAAAGGA
	Reverse	TGACACTTCCGTTAAAGCAGAAT
TRIB1	Forward	GCTGCAAGGTGTTTCCCATTA
	Reverse	TCCCCAAAGTCCTTCTCAAAGA
BCL6	Forward	GGAGTCGAGACATCTTGACTGA
	Reverse	ATGAGGACCGTTTTATGGGCT
TLR2	Forward	ATCCTCCAATCAGGCTTCTCT
	Reverse	GGACAGGTCAAGGCTTTTTACA
PTGS2	Forward	CTGGCGCTCAGCCATACAG
	Reverse	CGCACTTATACTGGTCAAATCCC
BCL3	Forward	CCGGAGGCGCTTTACTACC
	Reverse	TAGGGGTGTAGGCAGGTTCAC
IER3	Forward	CAGCCGCAGGGTTCTCTAC
	Reverse	GATCTGGCAGAAGACGATGGT
PLAUR	Forward	TGTAAGACCAACGGGGATTGC
	Reverse	AGCCAGTCCGATAGCTCAGG
CEBPD	Forward	GGAGAGACTCAGCAACGACC
	Reverse	TTGCGCTCCTATGTCCCAAG
MXD1	Forward	CGGGCTCATCTTCGCTTGT
	Reverse	GATTTGGTGAACGGCTTTTCTG
ACTB	Forward	TCGTGCGTGACATTAAGGAGAAGC
	Reverse	ATGGAGTTGAAGGTAGTTTCGTGGATG

**Table 2:** Top ten hub genes obtained by five algorithms of Cytohubba.

MNC	MCC	EPC	DMNC	Degree
RPS20	RPS20	RPS20	GFM1	RPS20
RPS6	RPS6	RPS6	PELP1	RPS6
RPS27 A	RPS18	RPS18	RPS17	RPS27 A
SNRPD2	RPL26	RPL26	CCT7	SNRPD2
RPL26	RPL11	RPL11	RPL24	RPL26
RPL11	RPLP0	RPLP0	RPS18	RPL11
RPL9	RPL9	RPL9	EEF1 A1	RPL9
RPS2	RPS2	RPS2	RPL26	RPS2
RPL3	RPL3	RPL3	RPLP0	RPL3
NHP2 L1	NHP2 L1	NHP2 L1	RPL9	NHP2 L1

**Table 3:** Demographic, clinical features, and laboratory data of all the participants.

Variables	AMI Group (*n* = 14)	Control (*n* = 8)	*p*-Value
Demographic features			
Age (years)	60.714 ± 3.010	60.571 ± 4.099	0.553
Male/Female	12/2	6/2	0.531
Cardiovascular risk factors			
Hypertension	6 (42.86%)	4 (50%)	0.746
Dyslipidemia	1 (7.14%)	0	NA
Diabetes mellitus	5 (35.71%)	1 (12.5%)	NA
Current smoking	8 (57.14%)	2 (25%)	0.145
Vital signs on admission			
SBP (mmHg)	121.50 (114.75–140.50)	120.00 (110.00–140.00)	0.868
DBP (mmHg)	75.000 (69.500–94.000)	72.000 (70.000–78.000)	0.868
Heart rate (bpm)	78.000 (73.500–87.000)	80.000 (75.000–84.000)	0.973
Echocardiographic finding			
LVEF (%)	52.500 ± 1.738	63.286 ± 1.848	0.001
Laboratory findings			
hs-cTnT (ng/mL)	9.930 (8.138–10.000)	0.007 (0.004–0.0100)	0.000
CKMB (U/L)	342.00 (210.25–457.25)	13.00 (9.00–15.00)	0.000
NT-pro-BNP (pg/mL)	682.10 (266.33–894.50)	68.00 (41.70–98.10)	0.002
TC (mmol/L)	5.024 ± 0.199	3.556 ± 0.215	0.001
TG (mmol/L)	1.740 (0.758–2.395)	2.280 (1.880–3.790)	0.082
LDL-C (mmol/L)	3.161 ± 0.155	1.451 ± 0.217	0.000
HDL-C (mmol/L)	1.060 (0.833–1.365)	0.860 (0.780–0.970)	0.188
Medications			
Aspirin	11 (78.57%)	0	NA
Clopidogrel	2 (14.29%)	0	NA
Ticagrelor	9 (64.29%)	0	NA
Statin	7 (50%)	0	NA
ACEI/ARB	2 (14.29%)	3 (37.5%)	0.211
ß blocker	3 (21.43%)	2 (25%)	0.848
CCB	6 (42.86%)	0	NA

SBP: systolic blood pressure; DBP: diastolic blood pressure; LVEF: left ventricular ejection fraction; hs-cTnT: high-sensitivity cardiac troponin T; CKMB: creatine kinase-MB; NT-pro-BNP: *n*-terminal pro-B-type natriuretic peptide; TC: total cholesterol; TG: triglyceride; LDL-C: low-density lipoprotein-cholesterol; HDL-C: high-density lipoprotein-cholesterol; ACEI: angiotensin converting enzyme inhibitors; ARB: angiotensin II receptor blockers; and CCB: calcium channel blockers.

## Data Availability

The mRNA expression dataset used in our study was downloaded from the Gene Expression Omnibus (GEO) under accession number GSE61144 (https://www.ncbi.nlm.nih.gov/geo/query/acc.cgi?acc=GSE, accessed on 5 June 2021).

## Supplementary Materials

The following are available online at https://www.mdpi.com/article/10.3390/jcdd9010030/s1: Table S1: List of the top 50 DEGs in GSE, Table S2: List of the top 50 DEGs in the brown module, and Figure S1: Data quality checking and normalization with log2 transformation.
