# Supplementary material for: The Identification and Validation of Hub Genes Associated with Acute Myocardial Infarction Using Weighted Gene Co-Expression Network Analysis
Source: J Cardiovasc Dev Dis. 2022 Jan 17;9(1):30. doi: 10.3390/jcdd9010030 (PMC8778825; doi:10.3390/jcdd9010030)
Supplement: Supplementary file 1 [file jcdd-09-00030-s001.zip › jcdd-1527644-Supplementary information.pdf]

Table S1. List of the top 50 DEGs in GSE61144

Gene	adj. p-value	p-value	t	B	logFC
C11orf46	0.000304	1.45E−08	−8.13778	9.51894	−0.341
CDC91L1	0.000304	1.73E−08	−8.06147	9.36057	−0.329
RBMX	0.000304	1.87E−08	−8.02808	9.29097	−0.354
MS4A7	0.00034	2.80E−08	−7.85511	8.92772	−0.844
EXOSC8	0.000426	5.06E−08	−7.60278	8.38946	−0.452
ACTR5	0.000426	5.50E−08	−7.56803	8.31457	−0.334
NRD1	0.000426	7.55E−08	7.434865	8.02585	0.566
FCRL3	0.000426	7.87E−08	−7.4176	7.98823	−0.949
LOC440737	0.000544	1.12E−07	−7.27136	7.66771	−0.793
C10orf86	0.000564	1.54E−07	−7.13885	7.37452	−0.429
PRPS1	0.000564	1.60E−07	−7.1222	7.3375	−0.367
ZNF529	0.000564	1.73E−07	−7.09107	7.26818	−0.256
PPIE	0.000564	1.79E−07	−7.07693	7.23666	−0.455
PTPRE	0.000564	1.85E−07	7.062361	7.20413	0.453
RPL9	0.000587	2.11E−07	−7.00928	7.08537	−0.465
MRPS21	0.000587	2.17E−07	−6.99807	7.06025	−0.462
RPS27A	0.000657	2.56E−07	−6.92977	6.90675	−0.565
G6PD	0.000693	2.96E−07	6.872063	6.77654	0.619
LOC168850	0.000705	3.19E−07	−6.84134	6.70703	−0.23
NIN	0.00078	3.83E−07	6.767409	6.53921	0.382
QIL1	0.00078	4.00E−07	−6.74909	6.49753	−0.422
MGC2463	0.00078	4.18E−07	−6.73202	6.45863	−0.402
RERE	0.00078	4.30E−07	6.720112	6.43146	0.516
CD44	0.00078	4.37E−07	−6.71335	6.41603	−0.339
DDX47	0.00078	4.57E−07	−6.6959	6.37618	−0.475
KIAA0372	0.00078	4.76E−07	−6.67885	6.33722	−0.435
LOC441246	0.00078	4.81E−07	−6.67504	6.3285	−0.697
ACAA2	0.000843	5.64E−07	−6.61111	6.18198	−0.462
PAK1	0.000843	5.71E−07	6.60585	6.16991	0.537
CD47	0.000843	5.71E−07	−6.60578	6.16976	−0.42
LOC349236	0.000844	5.89E−07	6.593434	6.14138	0.655
USP32	0.00086	6.18E−07	6.573954	6.09659	0.555
ADCY4	0.000874	6.55E−07	6.550814	6.04331	0.585
FLJ20272	0.000874	6.65E−07	−6.54513	6.0302	−0.403
C6orf166	0.000874	6.97E−07	6.526163	5.98648	0.429
DBP	0.000874	7.40E−07	−6.50198	5.93065	−0.215
NHP2L1	0.000874	7.79E−07	−6.4817	5.88377	−0.343
FAM36A	0.000874	7.79E−07	−6.48164	5.88364	−0.299
LOC645385	0.000874	7.87E−07	−6.47749	5.87404	−0.787
GZMK	0.000874	7.89E−07	−6.47627	5.87122	−1.52
WDR54	0.000908	8.39E−07	−6.45192	5.81483	−0.562
EXOSC10	0.000909	8.59E−07	−6.44263	5.7933	−0.392
AOF2	0.000944	9.11E−07	−6.41921	5.739	−0.367

MDC1	0.000971	9.67E−07	−6.39553	5.68401	−0.37
PCGF4	0.000971	9.77E−07	−6.39128	5.67412	−0.42
BCL7C	0.000981	1.01E−06	−6.37894	5.64544	−0.319
LOC149224	0.000985	1.03E−06	−6.36959	5.62368	−0.54
INPP5A	0.00101	1.08E−06	6.351901	5.5825	0.466
NADK	0.001038	1.13E−06	6.333399	5.53939	0.671
BCCIP	0.001062	1.18E−06	−6.31699	5.50112	−0.281

Table S2. List of the top 50 DEGs in the brown module

Gene	colorNEW	kMEblue	kMEbrown	kMEgrey	kMEturquoise
CCNDBP1	brown	0.677089	−0.87308	−0.7335	−0.51697
PDCD2	brown	−0.57421	0.810704	0.784412	0.482102
LOC649049	brown	−0.81599	0.934625	0.835439	0.741804
MADD	brown	−0.84944	0.770199	0.752435	0.813797
MGMT	brown	−0.52787	0.801289	0.718771	0.375899
FAM3C	brown	−0.70479	0.85749	0.817453	0.682185
DDX47	brown	−0.91749	0.909343	0.877926	0.860684
DDX39	brown	−0.7998	0.882686	0.851569	0.768285
LOC646630	brown	−0.72326	0.766	0.681324	0.643667
ASF1A	brown	−0.61507	0.867027	0.783575	0.525686
NRD1	brown	0.851168	−0.84976	−0.87703	−0.79535
NOL7	brown	−0.63956	0.78173	0.699455	0.476235
ZNF259	brown	−0.57411	0.77582	0.66697	0.427824
ADARB1	brown	−0.85744	0.831793	0.854621	0.879338
CD40	brown	−0.47305	0.743623	0.665612	0.362053
LOC221143	brown	−0.64181	0.837447	0.84531	0.645069
PDCD7	brown	−0.6816	0.884543	0.806868	0.577635
RPLP0	brown	−0.80812	0.819797	0.775202	0.802248
MGA	brown	−0.40538	0.782154	0.684436	0.281649
LOC648622	brown	−0.5577	0.864104	0.674433	0.300723
PSTPIP1	brown	0.675053	−0.77351	−0.5615	−0.36729
SLCO4C1	brown	0.809538	−0.8199	−0.75922	−0.72063
FBXL11	brown	0.869373	−0.83247	−0.77273	−0.74831
CPSF3	brown	−0.61802	0.774376	0.776723	0.580867
MYD88	brown	0.858522	−0.78293	−0.772	−0.7944
SOCS2	brown	−0.54009	0.725891	0.571341	0.299448
HMGN1	brown	−0.70215	0.864741	0.656478	0.420687
LOC642817	brown	−0.69155	0.920724	0.743657	0.469475
ATG9A	brown	0.73166	−0.74226	−0.77435	−0.7436
WWP1	brown	−0.69145	0.883762	0.831423	0.546634
CEBPZ	brown	−0.31656	0.691373	0.606854	0.117404
LOC347292	brown	−0.59695	0.874123	0.686718	0.35709
LOC644039	brown	−0.50708	0.85716	0.74351	0.327588
SETX	brown	0.803397	−0.72016	−0.69212	−0.78582
ZXDB	brown	−0.71733	0.81025	0.856452	0.747241
HOP	brown	−0.54878	0.792151	0.624609	0.302408

RPL3	brown	−0.8043	0.938947	0.848476	0.71594
RPS20	brown	−0.79405	0.952941	0.856749	0.683831
ZMYND11	brown	−0.48998	0.849861	0.698426	0.287815
LOC440733	brown	−0.86683	0.822515	0.818049	0.857784
DKFZP779L1558	brown	−0.54022	0.843115	0.698475	0.32388
STK19	brown	0.628639	−0.80286	−0.70032	−0.42464
KIAA1949	brown	0.681257	−0.75757	−0.6747	−0.44192
DC2	brown	−0.78499	0.809173	0.767472	0.774327
RPS27A	brown	−0.7438	0.925202	0.86605	0.640842
COX6C	brown	−0.88783	0.893342	0.827248	0.811088
DNM1L	brown	−0.59581	0.805104	0.736913	0.467038
RPL26	brown	−0.80152	0.868839	0.775376	0.682135
ZC3H8	brown	−0.55943	0.778325	0.791767	0.506286
MYL6	brown	0.800516	−0.83991	−0.79117	−0.70642

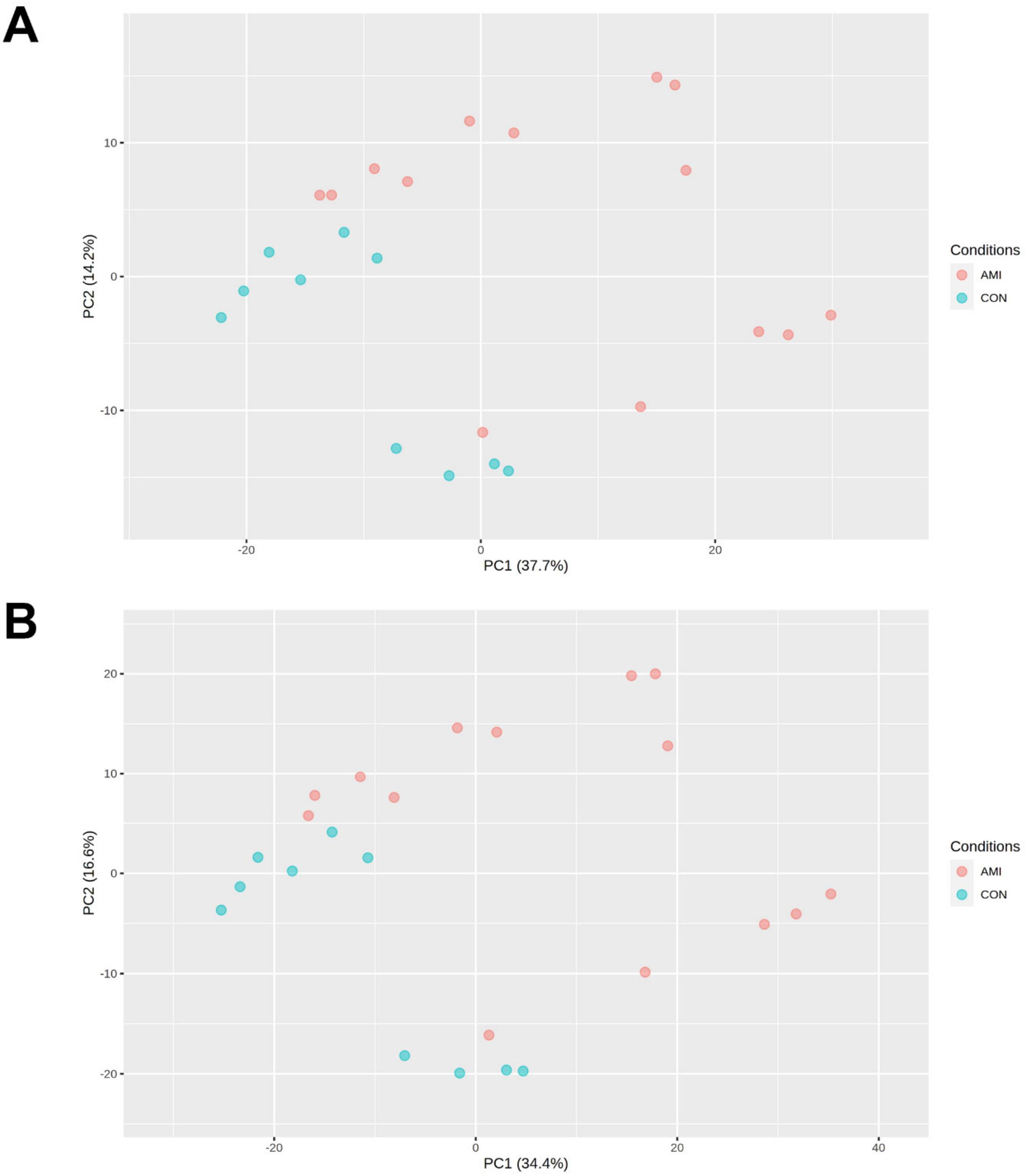

Figure S1. Data quality checking and normalization with log2 transformation. PCA plots before (A) and after (B) batch correction.
